# Supplementary material for: Toward a Universal Meta-Optics Solver via Large Language Models
Source: Nano Lett. 2026 Sep 3;26(36):12162–70. doi: 10.1021/acs.nanolett.6c03323 (PMC13587771; doi:10.1021/acs.nanolett.6c03323)
Supplement: Supplementary file 1 [file nl6c03323_si_001.pdf]

Supplementary Materials

Towards a universal meta-optics solver via large language models

Huanshu Zhang,[a] Lei Kang,[a] Yuyan Chen,[b] Luxiang Wang,[b] Zhaolong Cao,[b,+] Douglas H. Werner[a,*]

[a] The Pennsylvania State University, Department of Electrical Engineering, University Park, PA 16802, USA

[b] School of Electronics and Information Technology, Sun Yat-Sen University, Guangzhou 510275, China

This SI includes:

Supplementary Note 1. QNM simulation workflow
Supplementary Note 2. Additional information for dataset construction and preprocessing
Supplementary Note 3. MSE-distribution comparison for forward prediction
Supplementary Note 4. Balanced and unbalanced 8-family training

Supplementary Note 1. QNM simulation workflow

We employ the quasi-normal mode (QNM) method to fastly reconstruct three in-house-designed nanorod families, namely the 4rod, 3rod, and 2rod configurations, which consist of multiple silver nanorods embedded in a glass dielectric matrix [Phys. Rev. B **113**, 195435 (2026); Nanoscale **14**, 3849 (2022)]. Fundamentally, QNMs correspond to the resonant eigenmodes of open electromagnetic systems, obtained by solving the time-harmonic, source-free Maxwell's equations [Phys. Rev. B **97**, 205422 (2018)]:

$$\begin{bmatrix} 0 & i\varepsilon^{-1}(\boldsymbol{r},\tilde{\omega}_m)\nabla\times \\ -i\mu^{-1}(\boldsymbol{r},\tilde{\omega}_m)\nabla\times & 0 \end{bmatrix}\begin{bmatrix}\tilde{\boldsymbol{E}}_m(\boldsymbol{r}) \\ \tilde{\boldsymbol{H}}_m(\boldsymbol{r})\end{bmatrix} = \tilde{\omega}_m \begin{bmatrix}\tilde{\boldsymbol{E}}_m(\boldsymbol{r}) \\ \tilde{\boldsymbol{H}}_m(\boldsymbol{r})\end{bmatrix}.$$

Based on this framework, the full optical response of the designed metasurface can be rigorously reconstructed via a QNM field expansion:

$$\begin{bmatrix}\boldsymbol{E}_s(\boldsymbol{r},\omega) \\ \boldsymbol{H}_s(\boldsymbol{r},\omega)\end{bmatrix} = \sum_m \alpha_m(\omega)\begin{bmatrix}\tilde{\boldsymbol{E}}_m(\boldsymbol{r}) \\ \tilde{\boldsymbol{H}}_m(\boldsymbol{r})\end{bmatrix},$$

where $\boldsymbol{E}_s(\boldsymbol{r},\omega)$ and $\boldsymbol{H}_s(\boldsymbol{r},\omega)$ denote the scattered electric and magnetic fields of the system under external optical excitation, and $\tilde{\omega}_m$ represents the mth complex eigenfrequency of the QNM. In this work, the silver nanorod resonators are treated as nonmagnetic, with a permeability equal to the free-space permeability μ_0, while the homogeneous glass surrounding medium is characterized by a constant permittivity $\varepsilon_b = 2.25$. The frequency-dependent optical dispersion of silver is described using the classical Drude–Lorentz model, which is defined as:

$$\varepsilon(\omega) = \varepsilon_\infty\left(1 - \frac{\omega_p^2}{\omega^2 - \omega_0^2 + i\omega\gamma}\right),$$

where $\varepsilon_\infty, \omega_p, \gamma$ and ω_0 represent the high-frequency permittivity, plasma frequency, damping coefficient, and intrinsic resonant frequency of silver, respectively. To linearize the dispersive Maxwell operator and facilitate rigorous eigenmode analysis, two auxiliary field quantities, the polarization $\boldsymbol{P} = (\varepsilon - \varepsilon_\infty)\,\boldsymbol{E}$ and the current $\boldsymbol{J} = -i\omega\boldsymbol{P}$, are introduced within the nanorod resonant domains. With this linearization treatment, QNMs are strictly defined as the eigenmodes of the linearized, source-free Maxwell's equations subject to Sommerfeld radiation boundary conditions. Through elementary algebraic transformations, the original eigenvalue equation can be recast into a standard generalized linear eigenvalue problem:

$$\begin{bmatrix} 0 & -i\mu_0^{-1}\nabla\times & 0 & 0 \\ i\varepsilon_\infty^{-1}\nabla\times & 0 & 0 & -i\varepsilon_\infty^{-1} \\ 0 & 0 & 0 & i \\ 0 & i\omega_p^2\varepsilon_\infty & -i\omega_0^2 & -i\gamma \end{bmatrix} \begin{bmatrix} \tilde{\boldsymbol{H}}_m(\boldsymbol{r}) \\ \tilde{\boldsymbol{E}}_m(\boldsymbol{r}) \\ \tilde{\boldsymbol{P}}_m(\boldsymbol{r}) \\ \tilde{\boldsymbol{J}}_m(\boldsymbol{r}) \end{bmatrix} = \tilde{\omega}_m \begin{bmatrix} \tilde{\boldsymbol{H}}_m(\boldsymbol{r}) \\ \tilde{\boldsymbol{E}}_m(\boldsymbol{r}) \\ \tilde{\boldsymbol{P}}_m(\boldsymbol{r}) \\ \tilde{\boldsymbol{J}}_m(\boldsymbol{r}) \end{bmatrix}.$$

During spectral reconstruction, the optical response of individual silver nanorods is dominated by a single dominant QNM, which is numerically solved using COMSOL Multiphysics via the "Electromagnetic Waves, Frequency Domain" module. Perfectly matched layers (PMLs) are implemented at the upper and lower boundaries to satisfy the Sommerfeld radiation condition for open resonant systems, while periodic boundary conditions are applied to the remaining four lateral sides. Leveraging these precomputed QNMs, we reconstruct the optical responses of complex nanostructures consisting of 2, 3, and 4 coupled nanorod resonators, following the framework reported in Nanoscale **14**, 3849 (2022).

The proposed QNM-based reconstruction workflow exhibits excellent computational efficiency, requiring an average of only 12 seconds for single-spectrum reconstruction. For a comprehensive library of 130,000 2-4 rods metasurface configurations (Supplementary Figure 1), the total computational runtime is approximately 18 days. Beyond speedup, the QNM framework delivers exceptional reconstruction accuracy: the spectra recovered via the QNM expansion exhibit an MSE of 1.2e-3 relative to full-wave finite element simulation results.

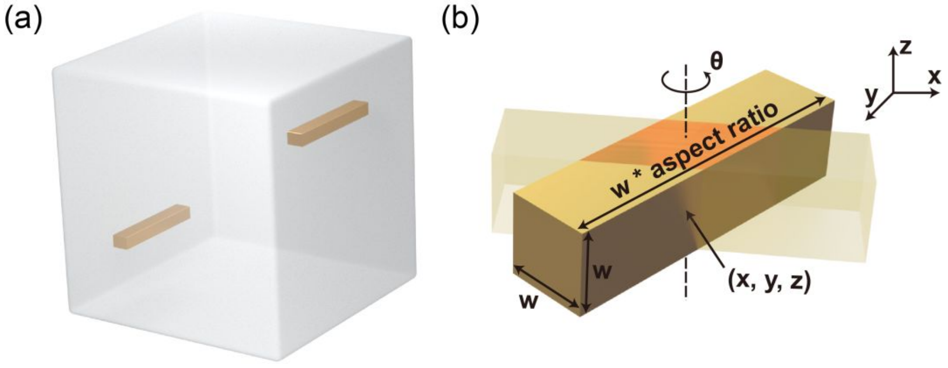

Supplementary Figure 1. Nanorod geometry in this work. (a) Representative coupled silver-nanorod structure embedded in a homogeneous glass dielectric matrix. The in-house rod families used in this work contain two, three, or four nanorods. (b) Parameterization of an individual nanorod. Each rod is described by a 6-element parameter vector: [width, aspect ratio, x, y, z, rotation angle], which specifies its center position (x, y, z), square cross-sectional width, aspect ratio, and in-plane rotation angle theta about the z axis. The rotation angle can only be 0 or $\frac{\pi}{2}$. The same coordinate convention and rod-level parameterization were used to construct the 2rod, 3rod,

and 4rod datasets. The final parameter vector describing the entire structure has 6n-3 elements, where the “-3” term indicates that the first rod position is fixed at (0, 0, 0) and all other rod positions are expressed relative to it.

Supplementary Note 2. Additional information for dataset construction and preprocessing

All geometry families were converted into a shared instruction-following JSON format before fine-tuning. For the forward task, each record contained the instruction, a geometry label, a wavelength-range string, an integerized parameter vector, and two integerized response arrays named T1 and T2. For the inverse task, the same fields were used with the input-output direction reversed: T1, T2, geometry, and wavelength were placed in the input, and the parameter vector was placed in the output.

An example of JSON format used in forward prediction:

```
{
  "instruction": "Provide the T1 and T2 of this metasurface.",
  "input": {
    "geometry": "AOPS",
    "wavelength": "1 - 1.8 um",
    "parameter": [44, 34, 46, 17, 21]
  },
  "output": {
    "T1": [225, 463, 591, …],
    "T2": [472, 325, 264, …]
  }
},
```

An example of JSON format used in inverse design:

```
{
  "instruction": "Provide a set of metasurface parameters that can produce this T1 and T2.",
  "input": {
    "geometry": "AOPS",
    "wavelength": "1 - 1.8 um",
    "T1": [225, 463, 591, …],
    "T2": [472, 325, 264, …]
  },
```

"output": {
"parameter": [44, 34, 46, 17, 21]
}
},

The shared names T1 and T2 were used as a modeling interface rather than as globally identical physical observables. Their physical meaning was family-specific and was retained through the geometry token and wavelength metadata. During evaluation, both target and predicted response arrays were divided by 1000 to restore their original magnitude before MSE calculation. The test set was never duplicated.

Supplementary Table 1. Dataset provenance, integer preprocessing, and balancing.

Family	Source	Definition of T1 and T2	Spectral Sampling	Parameter Vector and Integerization	Training/Test Counts and Balancing	Reported MSE	Our MSE
Cylinder	https://doi.org/10.1021/acsphotonics.9b00966	T1 = real part and T2 = imaginary part of the transmission coefficient.	Spectra were sampled every 5th point from the original grid, giving 61 values per channel over 5 - 10 um. Both channels were multiplied by 1000 and rounded to integers.	4 parameters. The first parameter was transformed by square root before scaling. All parameters were then multiplied by 1000 and rounded to integers.	52,488 loaded training records, of which 52,484 were unique JSON objects; 13,123 test records. The balanced 8-family forward file contains 104,976 cylinder training records, corresponding to exact x2 duplication of the loaded training file.	MSE = 3.5e-4 for real transmission and 2.3e-4 for imaginary transmission.	Combined MSE = 2.2e-3 for real and imaginary parts.
H	https://doi.org/10.1021/acsphotonics.9b00966	T1 = real part and T2 = imaginary part of the transmission coefficient.	Spectra were sampled every 5th point, giving 61 values per channel over 5 - 10 um. Both channels were multiplied by 1000 and rounded to integers.	6 parameters. The first parameter used in original paper was dropped as it doesn't change across entire dataset. The remaining parameters were multiplied by 1000 and rounded to integers.	19,840 training records and 4,960 test records. The balanced 8-family forward file contains 79,360 H records, corresponding to exact x4 duplication.	Not reported.	Combined MSE = 4.6e-3 for real and imaginary parts.
AOPS	https://doi.org/10.1038/s41598-024-72125-4	T1 = through-port transmission spectrum. T2 = drop-port transmission spectrum.	From 800 wavelength samples over 1 - 1.8 um, 41 equally spaced samples were retained. Both channels were multiplied by 1000 and rounded to integers.	5 parameters. The parameters provided were already in integer form.	117,964 training records and 29,492 test records. The balanced 8-family forward file contains 117,964 AOPS records, corresponding to no duplication.	Forward-model test loss/MSE = 0.03; Validation loss = 0.028	MSE = 4.1e-4.
Micropyramids	https://doi.org/10.1038/s41598-022-13832-8	T1 = transmission, T2 = reflection.	From 100 spectral points, 51 points were retained. Both channels were multiplied by 1000 and rounded to integers.	7 parameters. All parameters were multiplied by 1000 and rounded to integers. The wavelength string was sample-specific.	28,400 training records and 7,100 test records. The balanced 8-family forward file contains 113,600 micropyramid records, corresponding to exact x4 duplication.	Test MSE = 1.53e-4	MSE = 5.4e-4
Ellipse	https://doi.org/10.1109/ACCESS.2025.3552418	T1 = absorptivity spectrum, T2 was set to a zero vector of the same length.	Each channel contained 50 values over 0.857 - 2 um. T1 was multiplied by 1000 and rounded to integers.	14 parameters. Parameters were multiplied by 1000 and rounded to integers.	52,812 loaded training records, of which 52,598 were unique JSON objects; 999 test records. The balanced 8-family forward file contains 105,624 ellipse records, corresponding to exact x2 duplication of the loaded training file.	Best forward MSE = 6.39e-3	MSE = 2.5e-3
2rods	In-house silver-nanorod dataset	T1 = transmittance, T2 = reflectance	From the raw frequency grid, 41 points were retained and represented by the wavelength string 0.937 - 1.5 um. Both channels were multiplied by 1000 and rounded to integers.	9 parameters. The rod parameter vector uses 3 + (N - 1) x 6 values. Width for each rod was rounded as-is; AR was kept as an integer if already integer-valued, otherwise multiplied by 1000 and rounded; (x, y, z) were multiplied by 1e9 and rounded; Rotation angle was binary encoded as 0/1 from its two unique raw states: 0 or pi/2.	7,833 training records and 1,959 test records. The balanced 8-family forward file contains 62,664 records, corresponding to exact x8 duplication.	N/A	MSE = 4.8e-4
3rods	In-house silver-nanorod dataset	T1 = transmittance, T2 = reflectance	From the raw frequency grid, 41 points were retained and represented by the wavelength string 0.937 - 1.5 um. Both channels were multiplied by 1000 and rounded to integers.	15 parameters using the same rod-family rule described above.	55,697 training records and 13,925 test records. The balanced 8-family forward file contains 111,394 records, corresponding to exact x2 duplication.	N/A	MSE = 6.3e-4
4rods	In-house silver-nanorod dataset	T1 = transmittance, T2 = reflectance	From the raw frequency grid, 41 points were retained and represented by the wavelength string 0.937 - 1.5 um. Both channels were multiplied by 1000 and rounded to integers.	21 parameters using the same rod-family rule described above.	57,920 training records and 14,481 test records. The balanced 8-family forward file contains 115,840 records, corresponding to exact x2 duplication.	N/A	MSE = 9.2e-4

Reported MSE values are extracted from the corresponding source publications and are provided for context. In our work, MSE is calculated on the test set used here after the preprocessing listed in the table, after restoring the response values to their original scale, and using all retained values in both T1 and T2. Consequently, differences in spectral sampling, response-channel combination, train/test split, and metric definition can affect numerical comparability. We note that the MSE calculation intentionally included all-zero response channels for those families who have all-zero channels, because correctly learning that a particular response channel should remain zero is itself part of the prediction task for certain geometry families.

The source training JSON files contained 392,954 loaded records in total. Because small numbers of exact duplicate JSON objects were already present in the cylinder and ellipse source files, the unique-record count before the intentional balancing step was 392,736. This unique-record count is the "original training sample" count reported in the main text. Exact duplication for balancing produced 811,422 effective training records in the final 8-family balanced forward file. The merged test set contained 86,039 records and was not duplicated.

Supplementary Note 3. MSE-distribution comparison for forward prediction

Main-text Figure 2 shows representative forward-prediction spectra, but selected examples alone cannot indicate whether the joint 8-family model improves the overall error distribution. We therefore compared the full per-sample MSE distributions of the joint 8-family model with the corresponding single-family baselines for each geometry family (Supplementary Figure 2). For each test sample, MSE was computed after dividing both the target and predicted integer-valued T1 and T2 arrays by 1000, using all points in both response channels. The resulting per-sample MSE values were plotted on a log10 scale for each geometry family.

Supplementary Figure 2 shows that the joint 8-family model generally shifted the MSE distributions toward lower error relative to the single-family baselines. This trend was especially clear for H, AOPS, ellipse, 4 rods, and 2 rods, where the joint model reduced both the high-error tail and the central part of the distribution. For cylinder and micropyramids, the distribution-level changes were smaller, consistent with the more modest family-level improvements reported in the main text. These results support the interpretation that the spectra selected in Figure 2 are representative of broader test-set behavior rather than isolated favorable cases. True zero-MSE cases cannot be plotted directly on a log10 axis. For visualization only, zero-MSE values were merged into <1e-9 bars.

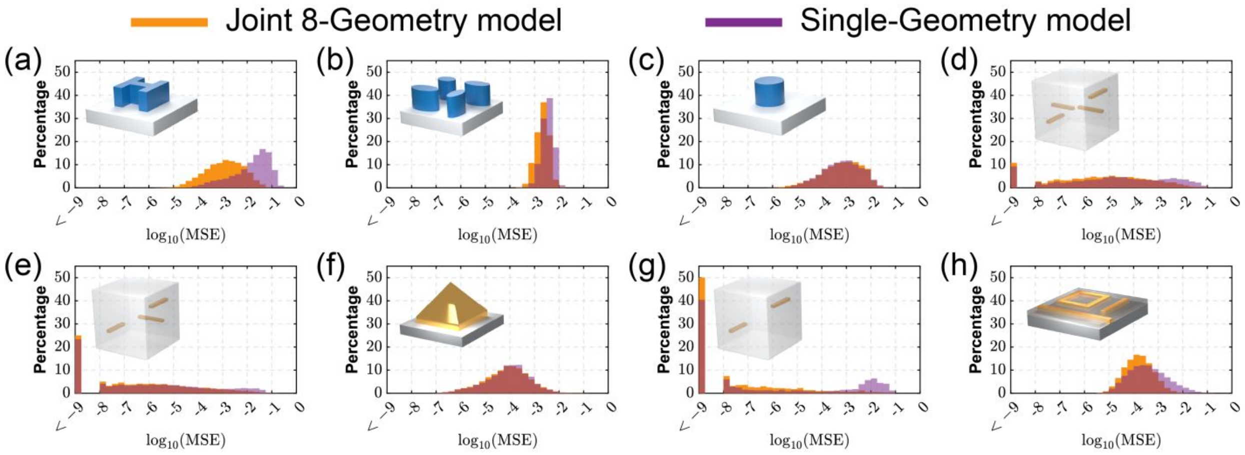

Supplementary Figure 2. Full test-set MSE distributions for single-family and joint 8-family forward models. Per-family histograms of log10(MSE) comparing the optimized single-family baseline model and the joint 8-family model. Purple histograms indicate single-family baselines, while orange histograms indicate the joint 8-family model. Histograms are normalized to percentages within each geometry family.

Supplementary Note 4. Balanced and unbalanced 8-family training

The balanced forward run used the balanced dataset, where minority families were oversampled by exact duplication according to Supplementary Table 1. The unbalanced control run used the unbalanced dataset, which retained the loaded source-family record counts without the intentional duplication step. Both runs used the same 8-family test set composition, the same base model, and the same fine-tuning hyperparameters. Supplementary Figure 3 shows the fine-tuning process and MSE comparison for each family.

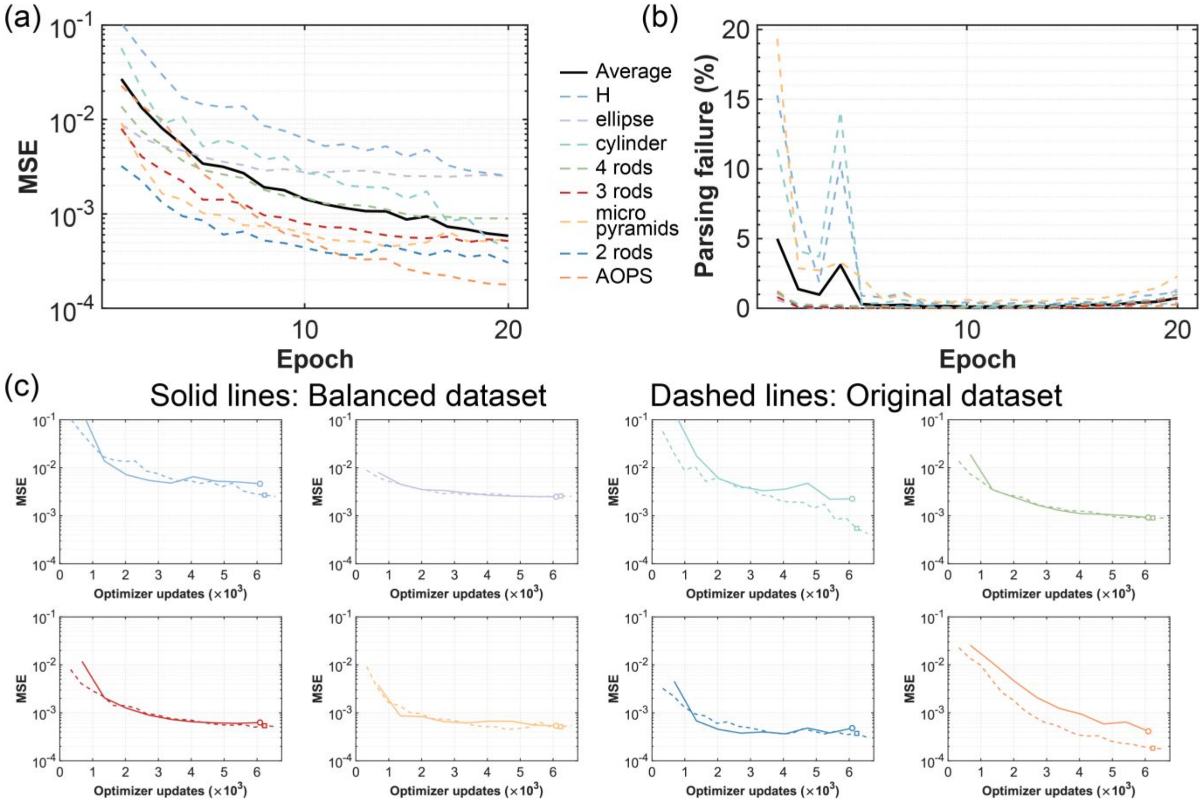

Supplementary Figure 3. Effect of exact-duplication balancing on 8-family forward fine-tuning. (a) Global MSE as a function of epoch for unbalanced training. (b) Global parsing failure rate as a function of epoch. (c) Family-resolved comparisons of MSE for the 8 geometry families. At the matched endpoints, the unbalanced run has a lower global MSE (6.20e-4 versus 1.09e-3) and lower MSE for 7 of 8 families, whereas the balanced run has a lower global parsing-failure rate (0.291% versus 0.471%). The same nominal 20-epoch linear-decay prescription was used in both runs, so the learning rates at the matched checkpoints differ because the runs have different total planned step counts.

To compare the runs at approximately matched exposure, we extended the unbalanced run to 19 epochs. Compared to epoch 9 of balanced fine-tune, this epoch differs by 2.28% in optimizer updates. At these approximately matched endpoints, the unbalanced run reached a lower global MSE (6.20e-4) than the balanced run (1.09e-3), corresponding to a 43.1% reduction relative to the balanced value. The unbalanced run also had lower MSE for 7 of the 8 families: AOPS (1.83e-4 unbalanced versus 4.13e-4 balanced), cylinder (5.45e-4 versus 2.25e-3), H (2.70e-3 versus 4.63e-3), micropyramids (5.15e-4 versus 5.38e-4), 2 rods (3.73e-4 versus 4.81e-4), 3 rods (5.39e-4 versus 6.28e-4), and 4 rods (8.99e-4 versus 9.19e-4). Ellipse was the only family for which the balanced checkpoint retained a lower MSE (2.48e-3 balanced versus 2.59e-3 unbalanced). The structured-output trend was different: the balanced run had a lower global parsing-failure rate at the matched endpoint (0.291% balanced versus 0.471% unbalanced). These results show that exact-duplication balancing changes the optimization trajectory and the tradeoff between numerical spectral error and structured-output reliability.
